# A rare case of atypical sympathetic ophthalmia post therapeutic keratoplasty

**DOI:** 10.1186/s12348-016-0104-1

**Published:** 2016-09-15

**Authors:** Vipul Bhandari, Sri Ganesh ᅟ, Mohan Raj, Akanksha Batra

**Affiliations:** 1 Nethradhama Superspeciality Eye Hospital, 256/14, Kanakapura Main Road, 7th Block, Jayanagar, Bangalore, Karnataka 560082 India; 2 Department of Vitreo-Retina, Nethradhama Eye Hospital, Kanakapura Road, Bangalore, 560070 India; 3 Department of Phaco-Refractive, Nethradhama Eye Hospital, Kanakapura Road, Bangalore, 560070 India

**Keywords:** Panuveitis, Sympathetic ophthalmia, Exudative retinal detachment

## Abstract

**Introduction:**

Sympathetic ophthalmia (SO) is a rare, bilateral, diffuse granulomatous uveitis that usually occurs after open globe injury or intraocular surgery.

**Methods:**

A patient developed SO following therapeutic penetrating keratoplasty (TPK) with cataract extraction in the exciting eye following fungal keratitis. The sympathizing eye presented with only posterior segment findings (exudative retinal detachment) and responded well with oral corticosteroids.

**Results:**

Graft remained clear in the left eye and the right eye; the best-corrected visual acuity (BCVA) improved to 0.2 log MAR.

**Conclusion:**

SO presenting after TPK for fungal keratitis is a rare occurrence but if detected early can be managed effectively.

## Introduction

Sympathetic ophthalmia (SO) has been defined as a “specific bilateral inflammation of the entire uveal tract of unknown etiology, characterised clinically by an insidious onset and a progressive course with exacerbation, and pathologically by a nodular or diffuse infiltration of the uveal tract with lymphocytes and epithelioid cells” [1].

## Case history

**Fig. 1:**
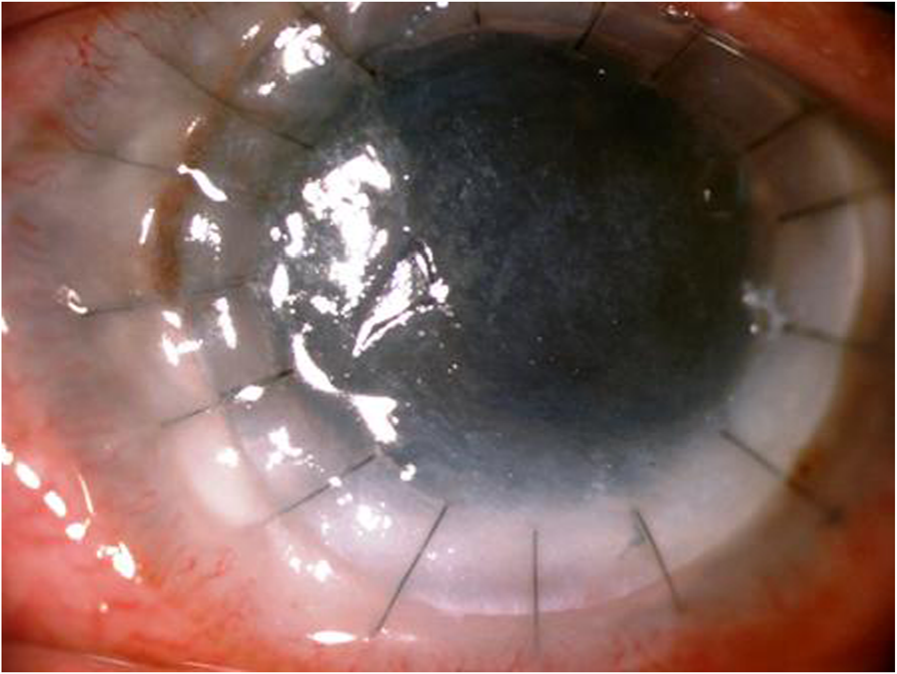
Clear graft following TPK

**Fig. 2:**
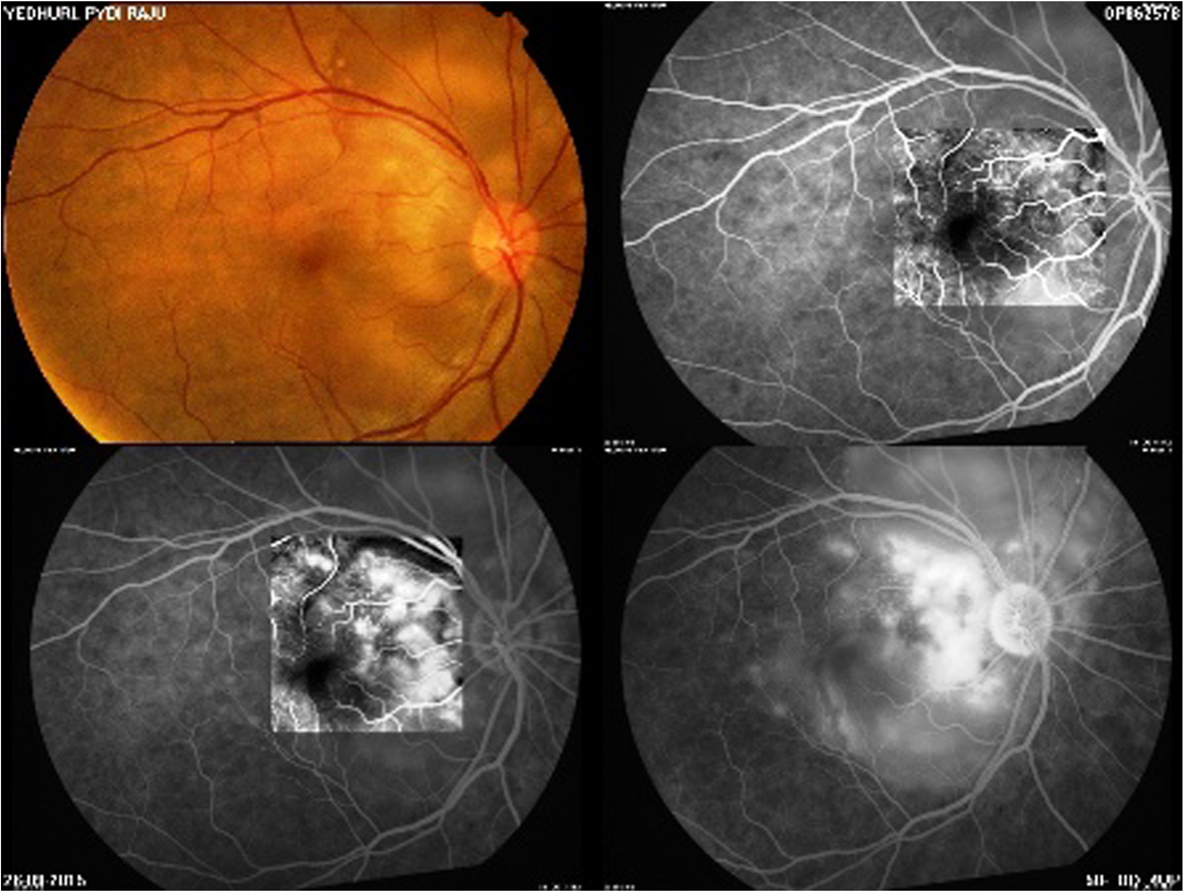
Pinpoint leakage on FFA in RE

A 48-year-old female presented to us with decrease in vision, redness and pain in the left eye (LE) 3 weeks after an injury with a stick. An informed consent was taken, and approval from the Nethradhama eye hospital review board was obtained. On examination, her best-corrected visual acuity (BCVA) was hand movement (HM+) in LE and 0 log MAR in the right eye (RE). A diagnosis of fungal ulcer in the LE was made after a thorough clinical examination and laboratory investigations which included potassium hydroxide mount (KOH), Sabouraud’s dextrose agar (SDA), and she was started on Natamet (natamycin 5 % Sun Pharma, India) eye drops hourly, Itral 1 % ointment (itraconazole, Java Pharma, India) three times a day, and Atropine eye ointment (Atropine 1 %, Java Pharma, India). The ulcer did not show any signs of improvement with medical treatments after 7 days, and accelerated cross-linking (KXL) was done in the LE with riboflavin 0.1 % solution (Vibex Rapid-Riboflavin 0.1 % with hydroxypropyl methylcellulose) applied every 2 min for 10 min before the irradiation. The cornea was then exposed to ultraviolet radiant energy from a solid-state UV lamp source (light-emitting diode (LED)) at a wavelength of 365–370 nm (in the UV-A spectral band) at an irradiance (“exposure dose rate”) of 30 mW/cm^2^ for 3 min to achieve a total radiant exposure (“total dose”) of 5.4 J/cm^2^. The ulcer still progressed, and a therapeutic penetrating keratoplasty (TPK) was done with cataract extraction as the lens was cataractous and postoperative antifungal therapy was continued. Posterior capsule was intact, and no intraocular lens was placed. Topical zymaxid eye drops (Gatifloxacin 0.5 %, Allergan, CA, USA) and flur eye drops (Flurbiprofen 0.03 %, Allergan, CA, USA) was started. Graft remained clear in the postoperative period (Fig. 1). Under the cover of anti-fungals, topical steroids were started at the end of 4 weeks, Lotepred eye drops (Loteprednol 0.3 %, Sun Pharma, India) was started with a frequency of four times per day. She presented with decrease in vision and floaters in the RE at the end of 3 months. On examination, BCVA in the RE was 0.4 log MAR and HM+ in the LE; on examination of the RE, the anterior chamber (AC) was optically clear, lens was clear but an exudative retinal detachment was seen on dilated fundus examination. Anterior segment optical coherence tomography (AS-OCT) and fundus fluorescein angiography (FFA) were done to confirm the diagnosis. FFA showed pinpoint leakage (Fig. 2), and AS-OCT showed an exudative retinal detachment. The LE did not show any AC reaction possibly due to use of topical steroids and showed no posterior segment changes. Patient was started on systemic corticosteroids at the dose of 1.5 mg/kg body weight for 2 weeks and then tapered to 1 mg/kg body weight with weekly tapering to a period of 6 weeks. Patients gradually responded to the treatment with resolution of exudative detachment and leakage on FFA and BCVA of 0.2 log MAR in the RE. Patient was started on systemic methotrexate 15 mg/kg body weight once a week with folic acid supplement after consulting an immunologist. BCVA is maintained in the RE, and graft is clear in the left eye.

## Discussion

Sympathetic ophthalmia occurs following penetrating injury or surgical procedures in one eye threatening sight in the fellow eye. Epidemiological estimates have shown the incidence to be 0.2 to 0.5 % after penetrating ocular injuries and 0.01 % after intraocular surgery [2,3].

The time from ocular injury to onset of SO varies from a few days to decades, with 80 % of the cases occurring within 3 months after injury to the exciting eye and 90 % within 1 year [4]. The classical description of signs include granulomatous mutton fat keratic precipitates, anterior chamber, and vitreous inflammation with or without yellow–white lesions in the retinal periphery. Other fundus lesions like retinal detachment, papillitis, optic atrophy, and vasculitis are reported uncommonly and are generally seen in conjunction with anterior segment inflammation [5]. Mcpherson et al. have reported two cases with atypical form of sympathetic ophthalmia with only posterior segment findings and termed it as “posterior sympathetic ophthalmia” [6]. The diagnosis of sympathetic ophthalmia is based on history and clinical examination. There are no specific laboratory studies to establish the diagnosis of SO; however, focused clinical testing can be used to rule out other disease entities with a similar clinical picture. Fluorescein angiography (FA) and indocyanine green video-angiography (ICG-V) are useful adjuncts in establishing the extent and severity of SO. Fluorescein angiography during acute sympathetic ophthalmia shows an exudative process and may provide evidence of multifocal areas of early hyperfluorescence (pinpoints) and leakage in the retinal pigment epithelium [1].Galor et al. found that although ocular complications were seen in many sympathizing eyes with SO, most patients maintained functional VA. The presence of an exudative retinal detachment and active intraocular inflammation correlated with poorer vision in the sympathizing eye [7].

A similar case of sympathetic uveitis after a tectonic corneal-scleral keratoplasty because of a fungal keratitis is reported by Magalhaes et al. where they studied the spectral-domain optical coherence tomographic changes in the sympathizing eye [8]. To our best knowledge, this is the first reported case of SO following TPK+cataract extraction following fungal keratitis with atypical SO manifesting only with posterior segment findings.
